# Arterial stiffness after 6 weeks postdelivery in women with a history of hypertensive disorders of pregnancy: a systematic review and meta-analysis

**DOI:** 10.3389/fmed.2025.1665100

**Published:** 2025-10-24

**Authors:** Xolani B. Mbongozi, Stuart D. R. Galloway, Angus M. Hunter, Jean Paul M. Milambo, Charles B. Businge

**Affiliations:** ^1^Department of Obstetrics and Gynaecology, Faculty of Health Sciences, Walter Sisulu University, Mthatha, South Africa; ^2^Faculty of Health Sciences and Sport, University of Stirling, Stirling, United Kingdom; ^3^School of Science and Technology, Clifton Campus, Nottingham Trent University, Nottingham, United Kingdom

**Keywords:** hypertensive disorders of pregnancy, arterial stiffness, cardiovascular disease, pulse wave velocity, augmentation index, preeclampsia, maternal morbidity, postdelivery risk

## Abstract

**Introduction:**

The main objective of this systematic review was to determine if the arterial stiffness remains elevated after 6 weeks post-delivery in women with a history of hypertensive disorders of pregnancy (HDP).

**Methods and analysis:**

A comprehensive systematic literature search was conducted across multiple electronic databases, including Medline, PubMed, Embase, Cochrane Library, Google Scholar, Web of Science, and CINAHL. We included studies assessing arterial stiffness in women with a history of HDP between 43 days and 10 years postdelivery, with participants under 60 years of age. The review followed the Preferred Reporting Items for Systematic Reviews and Meta-Analysis Protocols (PRISMA-P) guidelines. We extracted data on arterial stiffness indices, including carotid-femoral pulse wave velocity (cfPWV), augmentation index (AIx), and heart rate adjusted augmentation index (AIx@75), along with the mean ± standard deviation for each study. A random-effects model was used to pool data, and heterogeneity was explored through sensitivity and subgroup analyses. The Newcastle Ottawa Scale (NOS) and Grading of Recommendations Assessment, Development, and Evaluation (GRADE) were used to assess risk of bias and quality of evidence.

**Results:**

Out of 121 identified articles, 12 studies involving a total of 856 women were included in the final review after eliminating duplicates and irrelevant studies. The overall pooled data revealed a significantly elevated AIx and cfPWV among women with a history of HDP. Specifically, the mean difference (MD) in AIx was 11.63 (95% Confidence Interval (CI): [1.72–21.54]), and for cfPWV, the MD was 0.53 (95% CI: [0.27–0.78]). Notably, while AIx showed no significant change in women within 1 year postpartum (MD 14.85, 95% CI [−6.03–35.72]), an elevation was observed in those beyond 1 year post-delivery (MD 9.11, 95% CI [4.20–14.02]). cfPWV was also found to be elevated in HDP patients both within 1 year (MD 0.59, 95% CI [0.32–0.86]) and beyond 1 year (MD 0.45, 95% CI [0.03–0.88]). In cases of early-onset preeclampsia, AIx did not show a significant increase; however, a significant increase in cfPWV was observed, with AIx having an MD of 1.55 (95% CI: [−0.74–3.84]) and cfPWV an MD of 1.86 (95% CI: [0.25–3.47]). For late-onset preeclampsia, there was no significant difference in AIx (MD 2.44, 95% CI: [−8.82–13.70]) or cfPWV (MD 0.10, 95% CI: [−0.42–0.62]).

**Conclusion:**

This systematic review and meta-analysis suggest that arterial stiffness may remain elevated beyond 6 weeks postpartum in women with a history of HDP. However, the findings should be interpreted with caution due to heterogeneity across studies and limited number of available studies. Larger and standardized longitudinal studies are needed to confirm these results. In the meantime, regular cardiovascular monitoring for these women is recommended while awaiting more conclusive evidence.

**Systematic review registration:**

https://www.crd.york.ac.uk/PROSPERO/search, CRD42023461867.

## Introduction

Hypertensive disorders of pregnancy (HDP), including gestational hypertension and preeclampsia, contribute significantly to maternal morbidity and mortality worldwide (1). Research has consistently indicated that women with a history of HDP face an increased risk of long-term cardiovascular issues, such as hypertension, stroke, and heart disease (2, 3). One important marker of cardiovascular health is arterial stiffness, which has been observed to be elevated in women with HDP both during and after pregnancy (4, 5). Evidence suggests that arterial stiffness and central aortic blood pressure are more closely linked to future cardiovascular events and renal complications than peripheral blood pressure in non-pregnant populations, a trend that may extend to HDP women in terms of their long-term cardiovascular outcomes.

From a physiological standpoint, the six-week mark post-delivery is noteworthy as it typically signals the stabilization of hemodynamics (6, 7). Throughout pregnancy, women experience considerable metabolic demands and cardiovascular adaptations that differ by trimester and gradually normalize after delivery (8). Key cardiovascular changes during pregnancy include an increase in cardiac output and blood volume, accompanied by a decrease in systemic vascular resistance (SVR). In particular, during pregnancy, elevated serum levels of progesterone and relaxin, a peptide hormone produced by the corpus luteum and placenta, facilitate systemic vasodilation (7, 8). This process results in a 25 to 30% reduction in SVR during pregnancy, with levels normalizing within 2 weeks postpartum and other vascular changes returning to normal during the six-week puerperal phase (7). Examining how arterial stiffness evolves after this timeframe is essential for understanding a woman’s cardiovascular health trajectory and potential long-term risks.

Although studies indicate that central blood pressure and arterial stiffness measured before 14 weeks of gestation can predict preeclampsia (37–39), further evaluation is needed on their impact on future cardiovascular health. Importantly, increased maternal arterial stiffness in hypertensive pregnancies has also been associated with intrauterine growth restriction and small-for-gestational-age infants (9). Research has also found associations between elevated maternal arterial stiffness and central blood pressure, foetal growth restriction, and small for gestational age infants, as well as increased blood pressure in offspring by the age of 3 (9, 10). However, there is limited global data on whether arterial stiffness remains elevated beyond 6 weeks postpartum in women who have a history of HDP.

Global research examining this issue has included: (i) The United Kingdom indicates that women with a history of HDP face significant long-term cardiovascular risks, including accelerated cardiovascular aging and a greater variety of conditions such as valvular heart disease (11); (ii)Canada has shown a significant association between HDP and long-term cardiovascular risk, particularly highlighting that women who have experienced preeclampsia may exhibit increased arterial stiffness for up to 6 years after giving birth (12). This growing body of evidence suggests that arterial stiffness may remain elevated beyond puerperium, reinforcing the need for regular cardiovascular monitoring in women with a history of HDP; (iii) In Asia, particularly Korea, research suggest that elevated arterial stiffness seen in women with HDP during pregnancy may return to baseline levels after delivery (13). This variation in findings emphasizes the importance of conducting a global review to understand the long-term effects of HDP on arterial health and; (iv)In Africa, where HDP is a major cause of maternal mortality, research on the lasting cardiovascular effects of HDP is still emerging but gradually increasing. Some studies suggest that women with a history of HDP may contend with a greater risk of cardiovascular disease; however, further research is essential to evaluate the specific role of arterial stiffness after delivery (5, 14). Given the significant long-term cardiovascular risks associated with HDP, it is crucial to understand the duration of elevated arterial stiffness. This systematic review synthesized the existing literature to provide a better picture of the long-term effects of HDP on arterial health.

## Objective

The objective of this systematic review was to determine if the arterial stiffness remains elevated after 6 weeks post-delivery in women with a history of hypertensive disorders of pregnancy.

## Methods

This protocol was developed following the Preferred Reporting Items for Systematic reviews and Meta-Analysis protocols (PRISMA-P) 2015 Guidelines (15). A comprehensive search was conducted in Medline, PubMed, Embase, Cochrane Library, Google Scholar, Web of Science, and CINAHL. The following MeSH terms for database searches were used: (“Arterial stiffness” OR “central blood pressure” OR “Pulse wave velocity” OR “Augmentation index” OR “PWV” OR “Aix” OR “AIx@75”) AND (“Preeclampsia” OR “Eclampsia” OR “Gestational hypertension” OR “Chronic hypertension” OR “Hypertensive disorders of pregnancy” OR “pregnancy induced hypertension”) AND (“After postpartum” OR “after delivery” OR “after puerperium”). One example of the search strategies used is shown in Table 1.

**Table 1:** PUBMED search strategy.

Search	Search items	Hits
1	Arterial stiffness [tw] OR central blood pressure [tw] OR Pulse wave velocity [tw] OR Augmentation index [tw] OR PWV [tw] OR Aix[tw] OR AIx@75[tw]	21,613
2	Preeclampsia [tw] OR Eclampsia[tw] OR Hypertensive disorders of pregnancy [tw] OR pregnancy-induced hypertension [tw] OR Gestational hypertension [tw] OR Chronic hypertension [tw]	69,722
3	After postpartum [tw] OR after delivery [tw] OR after puerperium [tw]	25,178
4	#2 AND #3	2,098
5	#1 AND #4	23

### The exposure

The exposure was hypertensive disease during pregnancy or within 42 days post-delivery. The hypertensive diseases included preeclampsia, gestational hypertension, chronic hypertension, and chronic hypertension with superimposed preeclampsia. Preeclampsia was defined as persistent *de novo* hypertension that develops at or after 20 weeks of gestation, accompanied by either proteinuria or features of maternal organ or uteroplacental dysfunction. Features of maternal organ dysfunction include acute kidney injury (creatinine ⩾90 μmol/L or 1 mg/dL), liver involvement (elevated alanine aminotransferase or aspartate aminotransferase >40 IU/L) with or without right upper quadrant or epigastric abdominal pain, neurological complications (such as eclampsia, altered mental status, blindness, stroke, clonus, severe headaches, and persistent visual scotomata), and haematological complications (decreased platelet count <150,000/μL, disseminated intravascular coagulation, haemolysis). Uteroplacental dysfunction includes fetal growth restriction, abnormal umbilical artery Doppler waveform analysis, or stillbirth (16, 17). Gestational hypertension was defined as persistent *de novo* hypertension that develops at or after 20 weeks of gestation in the absence of features of preeclampsia. Chronic hypertension referred to high blood pressure predating the pregnancy or recognized at < 20 weeks of gestation. Chronic hypertension with superimposed preeclampsia was diagnosed when preeclampsia occurred in a pregnant woman who had pre-existing chronic hypertension.

### Inclusion criteria

We included randomized and non-randomized control trials, prospective and retrospective cohort studies, case–control studies, and cross-sectional studies performed in humans. The studies were included based on the following criteria:

1. Women with a history of HDP, aged under 60 years.
2. Studies reporting arterial stiffness indices (cfPWV, AIx, and AIx@75) at least 43 days postpartum.

There were no restrictions by the date of publication.

### Exclusion criteria

We excluded case series and reports, cost–benefit analyses, and qualitative research, as well as reviews, newspapers, books, conference abstracts, theses, commentaries, letters, editorials, and unpublished data. Animal and *in vitro* studies were also excluded. Studies that only assess arterial stiffness measurements during pregnancy, labour, and delivery or within 6 weeks post-partum were excluded.

### Comparator

The control group consisted of women under 60 years old with no history of HDP, cardiovascular diseases, renal failure, or diabetes.

### Data collection

Mendeley Reference Manager was used to store and manage searched items under the following headings in a separate table: Authors, publication year, title of the study, location of the study, participants ages, country, and main study findings. Two independent reviewers screened all the titles and abstracts using the predetermined inclusion and exclusion criteria. We followed PRISMA-P guidelines. Records identified as potentially eligible based on title, abstract, and full texts were obtained to screen. Where consensus on eligibility could not be achieved, a third review author was involved in the discussion. When the same cohort was reported in multiple articles, the study which contains the largest sample was selected. Data from selected studies were extracted and entered into a Microsoft Excel spreadsheet.

### Risk of bias

Two independent authors evaluated the methodological quality of all included studies. Where consensus on eligibility could not be achieved, a third reviewer was involved in the discussion. The Newcastle Ottawa Scale (NOS) and Grading of Recommendations Assessment, Development, and Evaluation (GRADE) were used to assess risk of bias and quality of evidence (18). Disagreements among the two authors was resolved by consultation with the other authors to reach a consensus. No studies were excluded based on the risk of bias assessment.

Potential sources of heterogeneity were first assessed using the I^2^ statistic (19). Values of 25% or less were classified as low, around 50% as moderate, and 75% or more as high (20). The subgroup and sensitivity analyses using RevMan 5.4 and NOS assessment scale were performed. Since all subgroups contained fewer than ten studies, a funnel plot was only created for those with six or more studies.

### Statistical analysis

Estimates of mean and ± SD for arterial stiffness indices (cfPWV, AIx, AIx@75) for women who had HDPs (for each subtype of HDP, as available) and normotensive women was obtained from the relevant studies. For studies where the estimates were reported as the median and interquartile range, approximate estimates of mean and ±SD were calculated using the available estimates of the median and, first quartile and third quartile. These data were summarized using a DerSimonian and Laird random-effects model (21). A separate meta-analysis was conducted for each hemodynamic indices of interest (cfPWV, AIx, AIx@75) for the combined outcome in those who had HDPs. For studies that provided data on the association between these hemodynamic variables and the outcome of interest, we meta-analyzed these effect measures using a random effects model. We performed a subgroup analysis for each subtype of HDP, as available.

## Results

### Study selection

**Figure 1:**
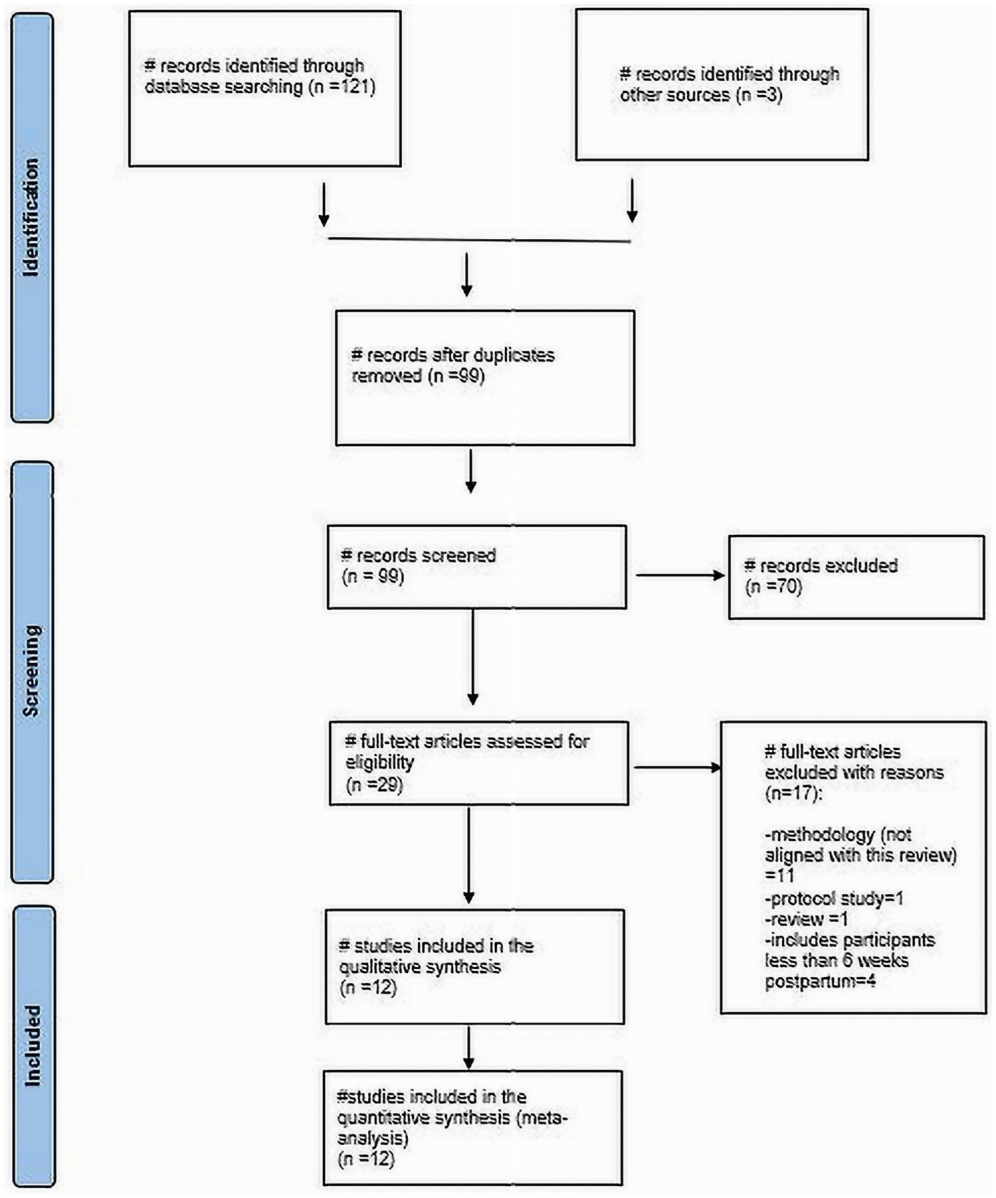
Flow diagram of study inclusion.

Through our literature search, we identified 121 articles. After removing 22 duplicates and excluding 70 based on title and abstract screening (see Figure 1), Twenty-nine articles remained that were eligible for full-text review. Out of these, 12 studies were included in the final review. Specifically, there were 4 cross-sectional studies, 5 cohort studies, and 3 case–control studies (Table 2). The total number of participants across the studies was 856 women with HDP and healthy women.

**Table 2:** Data extraction table.

Study	Authors (year)	Country	Study design	Sample size	Population characteristics (age, comorbidities)	Time postpartum	Arterial stiffness measure (cfPWV, AIx)	Mean ± SD of arterial stiffness	Comparison group	Elevated arterial STIFFNESS (Yes/No)	Risk of bias assessment (Cochrane/NOS)	Other key findings
1	Namugowa et al. (2017) (14)	South Africa	case-control	30 (15 preeclampsia 15 control)	African Black; 16–40 years; no CVD	15 weeks	cfPWV, AIx	Mean: 5.9 m/s ± 0.09. 23 ± 16%	Normal pregnancy group	Yes	Low risk	Significant difference in cfPWV between HDP and normal group
2	Pàez et al. (2009) (31)	Argentina	Case-control	40 (20;20)	18–33 years old; no CVD	2 years	cfPWV, AIx	Mean: 10.5 m/s ± 2.3; 37.5 ± 5.1%	Normal pregnancy group	Yes	Low risk	Significant difference in cfPWV between HDP and normal group
3	Christensen et al. (2017) (26)	Denmark	cohort	48 (24;24)	Average 40 years old; no CVD	1 year	cfPWV, AIx	Mean: 8.03 m/s ± 0.93; 22.1 ± 9.22%	Normal pregnancy group	No	Low risk	The hypertension exposed women tended to have a higher aPWV compared to unexposed women; but the difference was not significant
4	Polónia et al. (2014) (27)	Portugal	Cross-sectional	100 (45;55)	no CVD	10 years	cfPWV, AIx	6.7 m/s ± 1.1; 25.7 ± 5.1%	Normal pregnancy group	Yes	Low risk	Significant difference in cfPWV between HDP and normal group
5	Ehrenthal et al. (2014) (22)	United States of America	cohort	74 (33;41)	18 years and above; White and African Americans; no CVD or Diabetes	1 year	cfPWV, AIx	6.2 m/s ± 1.5; 22.0 ± 13.4;	Normal pregnancy group	Yes	Low risk	Significant difference in cfPWV between HDP and normal group
6	Franz et al. (2013) (24)	Austria	cohort	74 (53;21)	Unknown	3–6 months	cfPWV, AIx	9.9 ± 1.6; −10.7 ± 21.1	Normal pregnancy group	yes	Low risk	Presence of a higher cardiovascular risk in patients after early-onset pre-eclampsia.
7.	Werlang et al. (2023) (12)	Canada	Cross-sectional	80 (40;40)	Average 36 years; no CVD	6 months-6 years	cfPWV, AIx	6.3 m/s ± 1.0; 16.2 ± 2.5%	Normal pregnancy group	yes	Low risk	Significant difference in cfPWV between HDP and normal group
8.	Robb et al. (2009) (33)	United Kingdom	cohort	37 (22;15)	Average 30 years; no CVD	7 weeks	cfPWV	7.3 m/s ± 0.3	Normal pregnancy group	Yes	Low risk	Significant difference in cfPWV between HDP and normal group
9	Evans et al. (2011) (30)	United States of America	cohort	68 (18;50)	18–40 years Black and White participants; no preexisting cardiac disease or diabetes mellitus	16 months after delivery	cfPWV	cfPWV Mean: 2.39 m/s ± 0.8	Normal pregnancy group	No	Low risk	No significant difference in AIx between HDP and normal group
10	Moe et al. (2020) (23)	Norway	Cross-sectional	221 (126;95)	18 years and above; no CVD or renal disease	1 year after delivery	cfPWV	6.3 m/s ± 2.9	Normal pregnancy group	Yes	Low risk	Significant difference in AIx between HDP and normal group
11	Usselman et al. (2020) (18)	United States of America	Cross-sectional	24(12;12)	White, Hispanic, black women; no CVD	6–24 months after delivery	cfPWV	7.1 m/s ± 1.1	Normal pregnancy group	yes	Low risk	Significant difference in AIx between HDP and normal group
12	Orabona et al. (2017) (25)	Italy	Case–control	60 (30;30)	No CV risk factors, smoking, dyslipidemia, overweight, diabetes mellitus or chronic hypertension.	6 months–4 years after delivery	cfPWV	cfPWV Mean: 8.42 m/s ± 1.92	Normal pregnancy group	yes	Low risk	Significant difference in cfPWV between HDP and normal group

### Study characteristics

The included studies, conducted between 2009 and 2023, came from various countries: South Africa (1), Argentina (1), Denmark (1), Portugal (1), United States (3), Austria (1), Canada (1), the United Kingdom (1), Norway (1) and Italy (1) (see Table 2). These studies evaluated arterial stiffness, with some utilizing carotid-femoral pulse wave velocity (cfPWV) and/or augmentation index (AIx) and/or Augmentation index adjusted for heart rate (AIx@75).

HDP encompasses various types (16). While only two studies, Ehrenthal et al. (22) and Moe et al. (23), examined composite HDP, other studies focused on specific types, including preeclampsia, early preeclampsia, and late-onset preeclampsia. As a result, the findings were categorized into four groups: composite HDP, preeclampsia, early preeclampsia, and late-onset preeclampsia.

### Risk of bias assessment

The risk of bias was assessed using the NOS for cohort and case–control studies, and a modified NOS for cross-sectional studies. The assessment indicated that all studies had a low risk of bias (Tables 3, 4). Recognizing that NOS evaluates risk of bias but does not fully capture the overall certainty of evidence, the GRADE framework was further applied. This comprehensive assessment, which considers factors such as consistency, directness, and precision, rated the overall certainty of the evidence as moderate to high (Table 5).

**Table 3:** NOS for the risk of bias and quality assessment of NRSs.

Author Year	Selection				Comparability	Exposure	Selection	Nonresponse rate	Total Score
	Adequate definition of patient cases	Representativeness of patient cases	Selection of controls	Definition of controls	Control for important or additional factors	Ascertainment of exposure	Same method of ascertainment for participants		
Namugowa et al. 2019 (32)	⋆	⋆		⋆	⋆⋆	⋆	⋆		7
Christensen et al. 2017 (26)	⋆	⋆		⋆	⋆⋆	⋆	⋆	⋆	8
Robb et al. (2009) (33)	⋆	⋆		⋆	⋆⋆	⋆	⋆		7
Orabona et al. (2017) (25)	⋆	⋆		⋆	⋆⋆	⋆	⋆		8
Pàez et al. (2008) (31)	⋆	⋆		⋆	⋆⋆	⋆	⋆		7
Ehrenthal et al. (2014) (22)	⋆	⋆		⋆	⋆⋆	⋆	⋆		7
Franz et al. (2013) (24)	⋆	⋆		⋆	⋆⋆	⋆	⋆		7
Evans et al. (2011) (30)	⋆			⋆	⋆⋆	⋆⋆	⋆		8

NOS, Newcastle-Ottawa scale; NRS, non-randomized study.

Each study is evaluated across three domains:

- Selection of the study population (maximum: 4 stars).

- Comparability of groups (maximum: 2 stars).

- Exposure (Ascertainment of exposure, Same method of ascertainment for participants and Nonresponse rate)(maximum: 3 stars).

A star (⋆) indicates the score, or criteria met.

**Table 4:** NOS (adapted for cross-sectional studies) for the risk of bias and quality assessment of NRSs.

Author Year	Selection					Comparability	Exposure	Total score
	Representativeness of the sample	Sample size	Non-respondents	Ascertainment of the exposure (risk factor)	Control for important or additional factors	Assessment of the outcome	Statistical test	
Polonia et al. (2014) (27)	⋆		⋆	⋆⋆	⋆⋆	⋆⋆	⋆	9
Werlang et al. (2023) (12)	⋆		⋆	⋆	⋆⋆	⋆⋆	⋆	8
Moe et al. (2020) (23)								7
Usselman et al. (2020) (18)	⋆		⋆	⋆	⋆⋆	⋆⋆	⋆	8

NOS, Newcastle-Ottawa scale; NRS, non-randomized study.

Each study is evaluated across three domains:

- Selection of the study population (maximum: 4 stars).

- Comparability of groups (maximum: 2 stars).

- Outcome (for cohort studies) or Exposure (for case-control studies) (maximum: 3 stars).

A maximum score of 9 stars represents the highest quality, indicating the lowest risk of bias. A star (⋆) indicates the score, or criteria met.

**Table 5:** Risk of bias assessment using NOS and GRADE for included studies (case-control and cross-sectional designs).

Author	Year	Study design	Selection	Comparability	Exposure/Outcome	Total NOS score	GRADE quality
Namugowa et al.	2019	Case-Control	3 ⋆	2 ⋆⋆	2 ⋆	7	Moderate
Christensen et al.	2017	Case-Control	3 ⋆	2 ⋆⋆	3 ⋆	8	Moderate to High
Robb et al.	2009	Case-Control	3 ⋆	2 ⋆⋆	2 ⋆	7	Moderate
Orabona et al.	2017	Case-Control	3 ⋆	2 ⋆⋆	3 ⋆	8	Moderate to High
Pàez et al.	2008	Case-Control	3 ⋆	2 ⋆⋆	2 ⋆	7	Moderate
Ehrenthal et al.	2014	Case-Control	3 ⋆	2 ⋆⋆	2 ⋆	7	Moderate
Franz et al.	2013	Case-Control	3 ⋆	2 ⋆⋆	2 ⋆	7	Moderate
Evans et al.	2011	Case-Control	2 ⋆	2 ⋆⋆	3 ⋆⋆	8	Moderate to High
Polonia et al.	2013	Cross-Sectional	2 ⋆	2 ⋆⋆	3 ⋆⋆ + ⋆	9	High
Werlang et al.	2023	Cross-Sectional	2 ⋆	2 ⋆⋆	3 ⋆⋆ + ⋆	8	Moderate to High
Moe et al.	2020	Cross-Sectional	1	2	2	7	Moderate
Usselman	2020	Cross-Sectional	2 ⋆	2 ⋆⋆	3 ⋆⋆ + ⋆	8	Moderate to High

Each study is evaluated across three domains:

- Selection of the study population (maximum: 4 stars).

- Comparability of groups (maximum: 2 stars).

Outcome (for cohort studies) or Exposure (for case-control studies) (maximum: 3 stars).

A maximum score of 9 stars represents the highest quality, indicating the lowest risk of bias. A star (⋆) indicates the score, or criteria met.

### Comparison of augmentation index and pulse wave velocity between women with all hypertensive disorders of pregnancy and normotensive women

**Figure 2:**
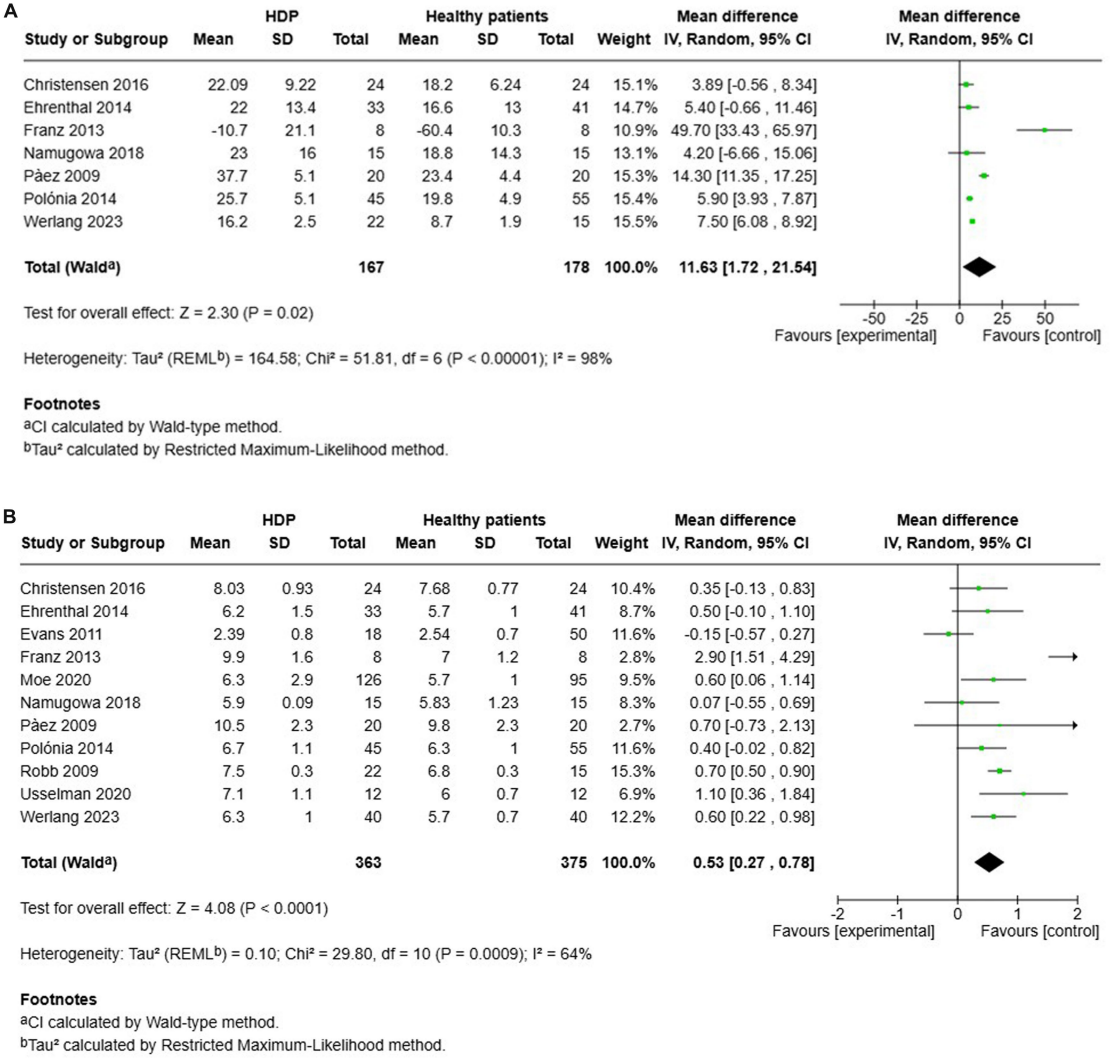
The pooled mean difference of the AIX **(A)** and PWV **(B)** of women with various HDP and normotensive women.

**Figure 3:**
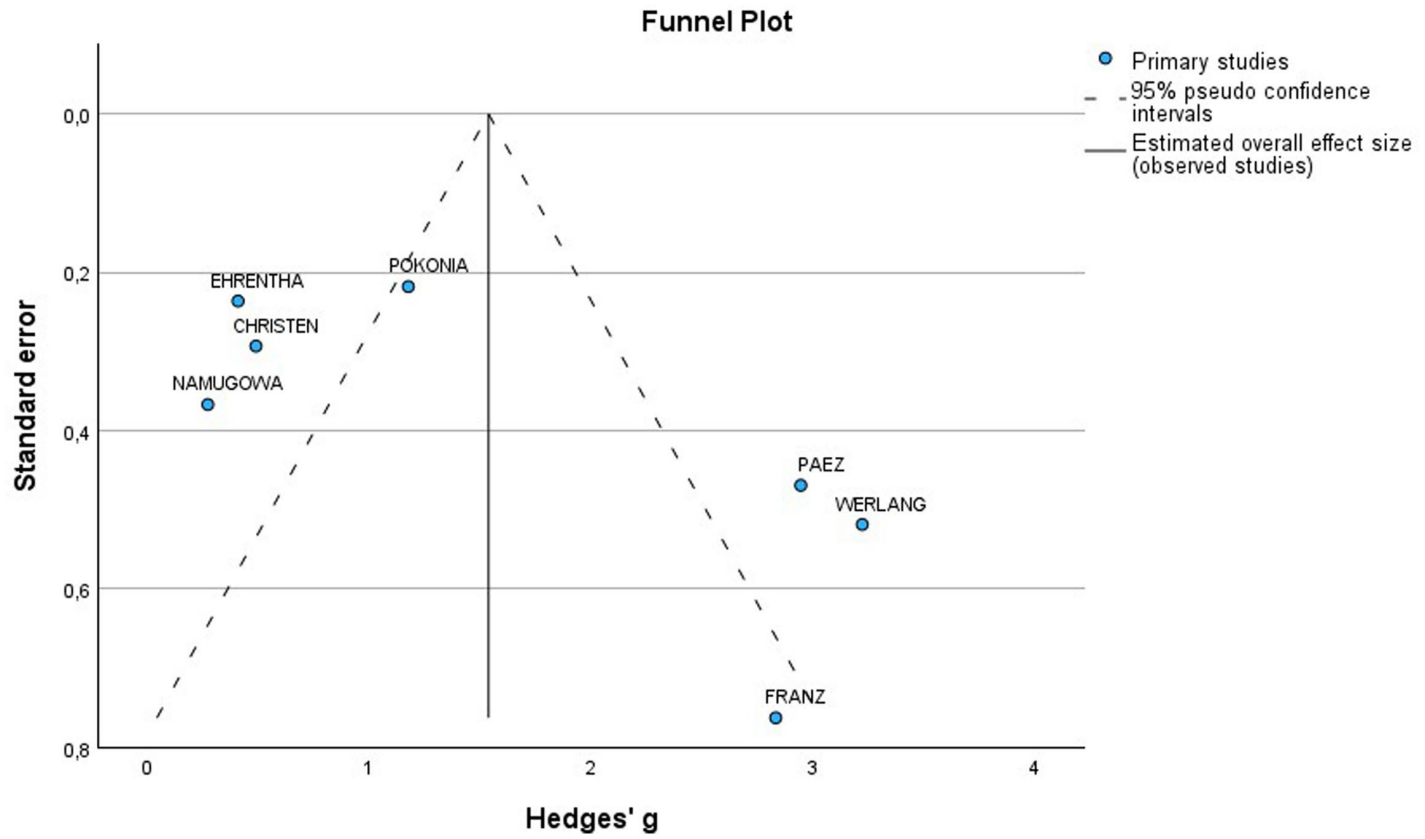
Funnel plot of included studies for AIx in women with HDP vs normotensive patients.

A total of 7 studies assessed the AIx in patients with all various HDP compared to normotensive women (Figure 2A). The pooled data demonstrated a significantly elevated AIx among women with HDP, indicating a Mean difference (MD) of 11.63 and a 95% confidence interval (CI) of [1.72–21.54]. This analysis included 345 participants, comprising 167 women with HDP and 178 normotensive controls. The heterogeneity among the studies was high, with an I^2^ value of 98%. Additionally, the analysis of funnel plots indicated the presence of publication bias, with a *p* value of 0.045 in the Egger’s test (Figure 3).

**Figure 4:**
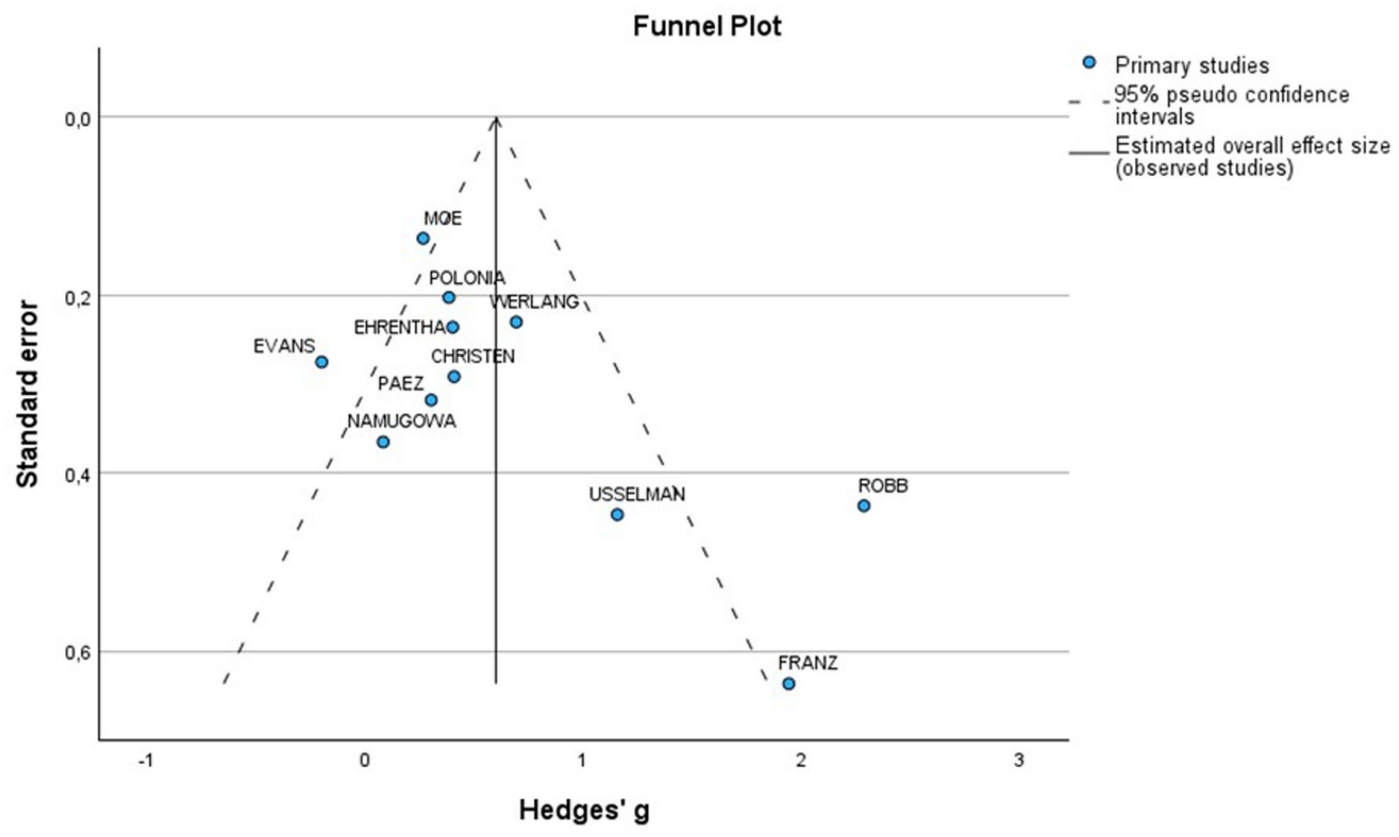
Funnel plot of included studies for PWV in women with HDP vs normotensive patients.

Furthermore, a total of 11 studies examined cfPWV in women with all various HDP compared to normotensive women (Figure 2B). The pooled data showed a significantly elevated cfPWV in women with HDP, with a MD of 0.53 and a 95% CI of [0.27–0.78]. This analysis included 738 participants, comprising 363 women with HDP and 375 normotensive controls. The studies showed moderate heterogeneity, with an *I*^2^ value of 64%. Additionally, the analysis of funnel plots indicated the presence of publication bias, with a *p* value of 0.002 in the Egger’s test (Figure 4).

### Comparison of augmentation index and pulse wave velocity between women with hypertensive disorders of pregnancy and normotensive women, within a year after delivery and after more than a year after delivery

**Figure 5:**
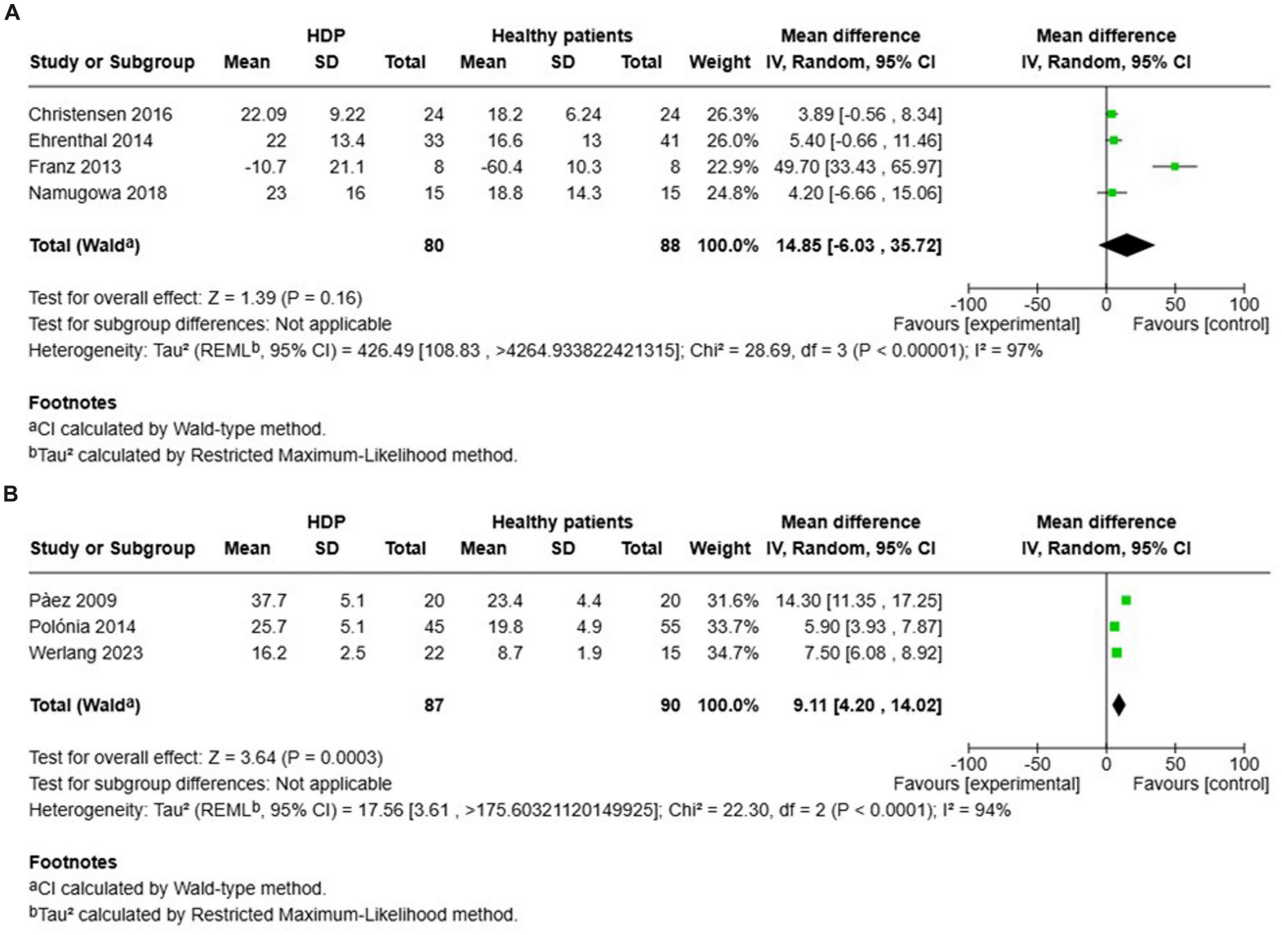
Pooled mean difference of the AIX of women with HDP and normotensive pregnant women, within a year or less after delivery **(A)**, and Pooled mean difference of the AIX of women with HDP and normotensive pregnant women after one year following delivery **(B)**.

Four studies investigated AIx in patients with HDP compared to normotensive controls within 1 year after delivery (see Figure 5A). The results showed no significant increase in AIx for women with HDP, reporting a MD of 14.85 and a 95% CI of [−6.03–35.72]. Additionally, there was high heterogeneity among these studies, with an *I*^2^ value of 97%.

In contrast, other studies focused on AIx in patients with HDP compared to normotensive controls more than 1 year after delivery (see Figure 5B). The findings indicated a significant increase in AIx for women with HDP, with a MD of 9.11 and a 95% CI of [4.20–14.02]. The heterogeneity in these studies was also high, with an *I*^2^ value of 94%.

**Figure 6:**
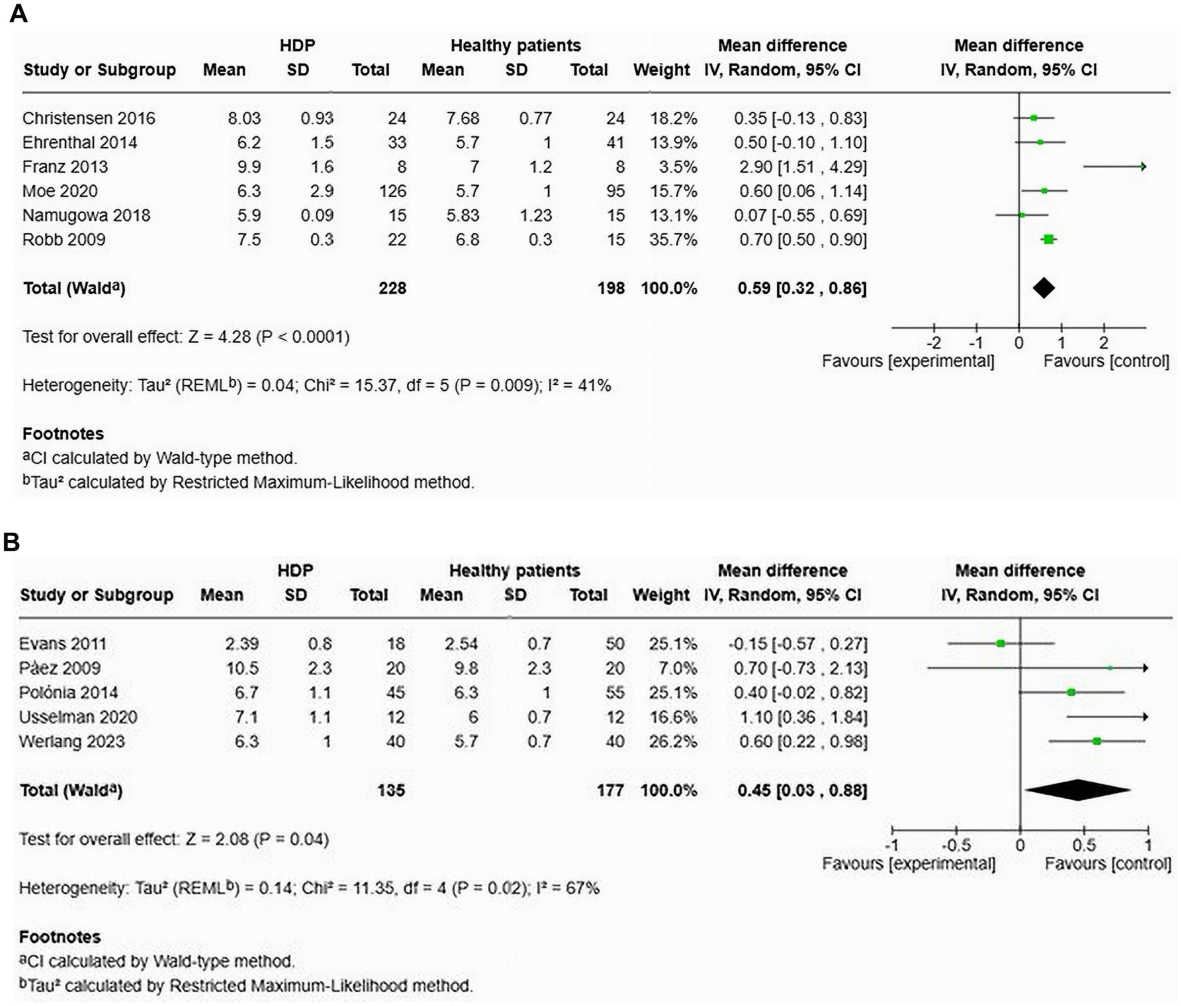
Pooled mean difference of the PWV of women with HDP and normotensive pregnant women, within a year or less after delivery **(A)**, and Pooled mean difference of the PWV of women with HDP and normotensive pregnant women after one year following delivery **(B)**.

Six studies evaluated cfPWV in women with HDP compared to normotensive controls within 1 year after delivery (Figure 6A). The results demonstrated a significantly higher cfPWV for women with HDP, reporting an MD of 0.59 and a 95% CI of [0.32–0.86]. The heterogeneity among these studies was moderate, with an I^2^ value of 41%.

Additionally, five studies focused on cfPWV in women with HDP compared to normotensive controls more than 1 year after delivery (Figure 6B). The findings also revealed a significant increase in cfPWV for women with HDP, with an MD of 0.45 and a 95% CI of [0.03–0.88]. Heterogeneity in these studies was also moderate, with an *I*^2^ value of 67%.

### Comparison of augmentation index and pulse wave velocity in preeclamptic versus normotensive women

**Figure 7:**
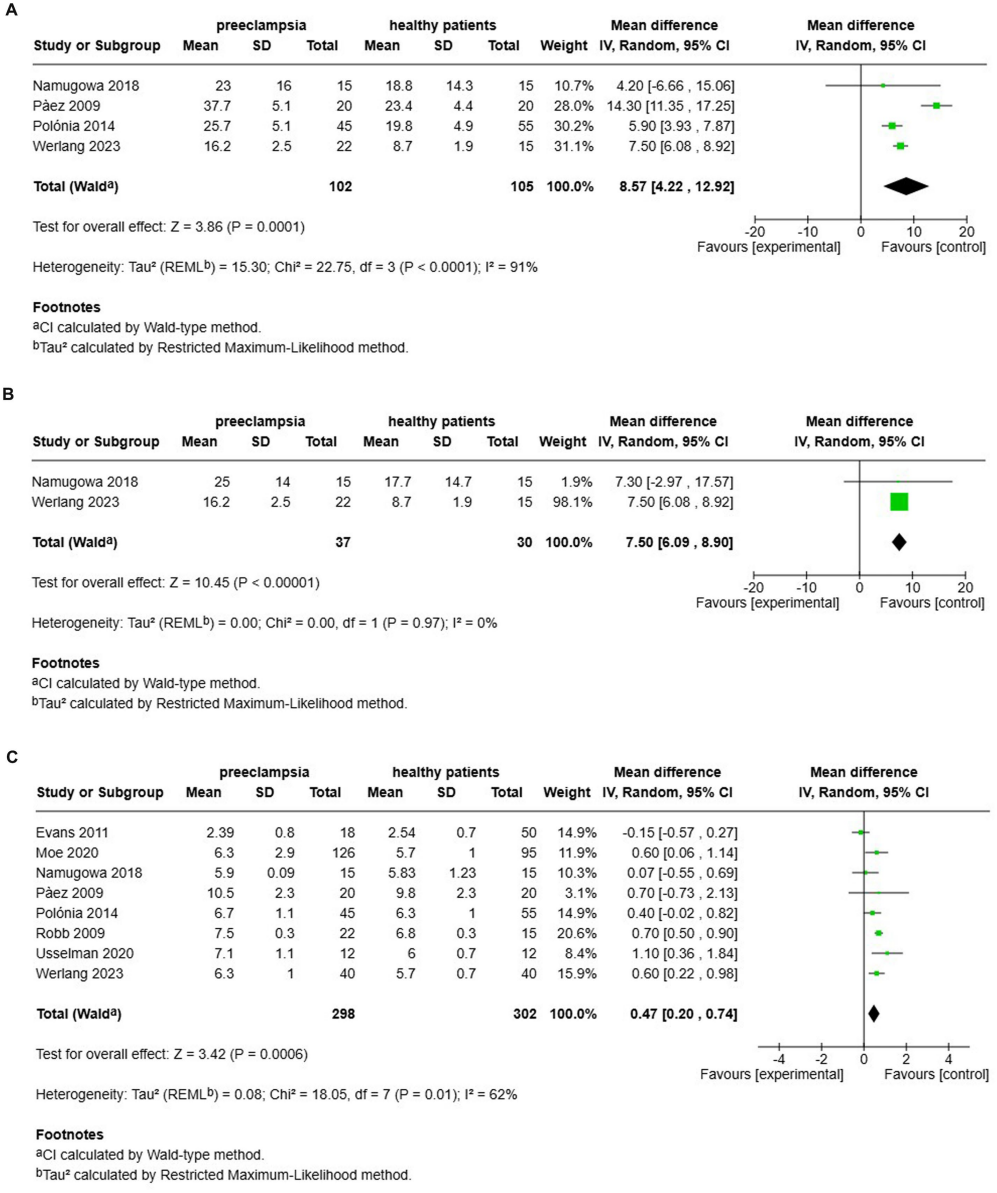
The pooed mean difference of the AIx **(A)**, AIx@75 **(B)** and PWV **(C)** of women with preeclampsia and normotensive women.

A total of 4 studies assessed the AIx in patients with preeclampsia compared to normotensive women (Figure 7A). This pooled data demonstrated a significantly elevated AIx among women with preeclampsia, with a mean difference (MD) of 8.57 and a 95% confidence interval (CI) of [4.22–12.92]. The analysis included 207 participants, comprising 102 women with preeclampsia and 105 normotensive controls. The heterogeneity among the studies was high at 91%, indicating varying results across the studies. In two studies (12, 14) that adjusted the AIx for heart rate (AIx@75), the augmentation index remained significantly elevated after the adjustment, with a MD of 7.50 and a 95% CI of [6.09–8.90] (Figures 7A,B). These two studies showed no heterogeneity.

Additionally, an examination of cfPWV across eight studies indicated a significant increase in cfPWV among women with preeclampsia, demonstrating an MD of 0.47 with a 95% CI of [0.20–0.74] (Figure 7C). This analysis involved 600 patients (298 preeclamptic and 302 normotensive). The studies displayed moderate heterogeneity, with an *I*^2^ value of 62%.

**Figure 8:**
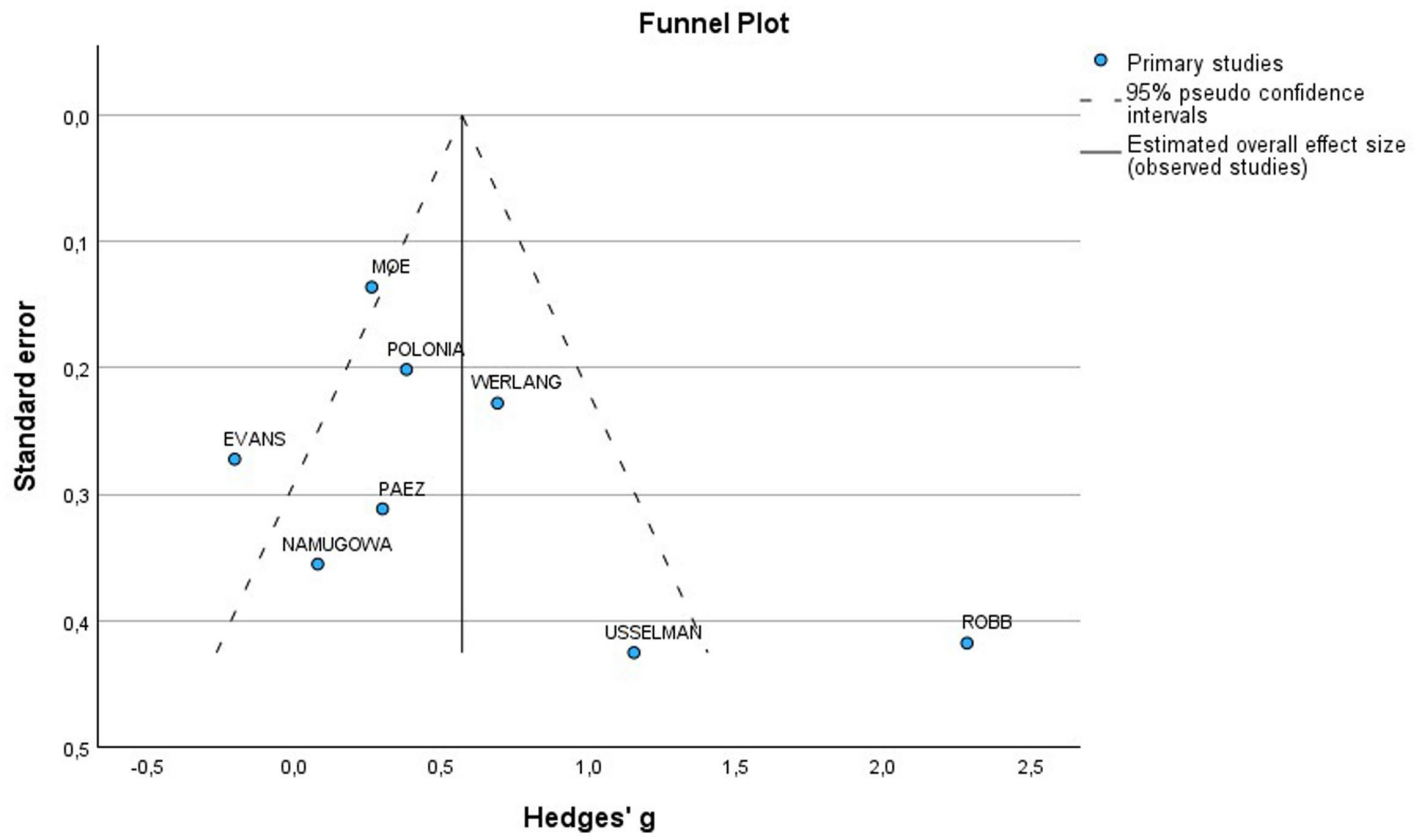
Funnel plot of included studies for PWV in preeclamptic vs normotensive patients. SE, Standard Error; MD, Mean difference.

The funnel plot was generally symmetrical; however, a few studies deviated from the main axis, indicating the possibility of a small sample effect or publication bias. To further investigate potential bias, Egger’s test was conducted, which showed no significant publication bias (*p* = 0.144) (Figure 8).

### Comparison of augmentation index and pulse wave velocity in women with HDP versus normotensive women

One study focused on AIx in patients with composite HDP against normotensive controls (22). The results indicated no significant increase in AIx for women with HDP (MD 5.40, 95% CI [−0.66–11.46]). The heterogeneity assessment was not applicable due to the design being based on a single study. This analysis included a total of 74 participants, with 33 diagnosed with HDP and 41 who were normotensive. However, AIx was found to be significantly elevated after adjusting for heart rate (AIx@75) (MD 6.40, 95% CI [0.52–12.28]) (15).

**Figure 9:**
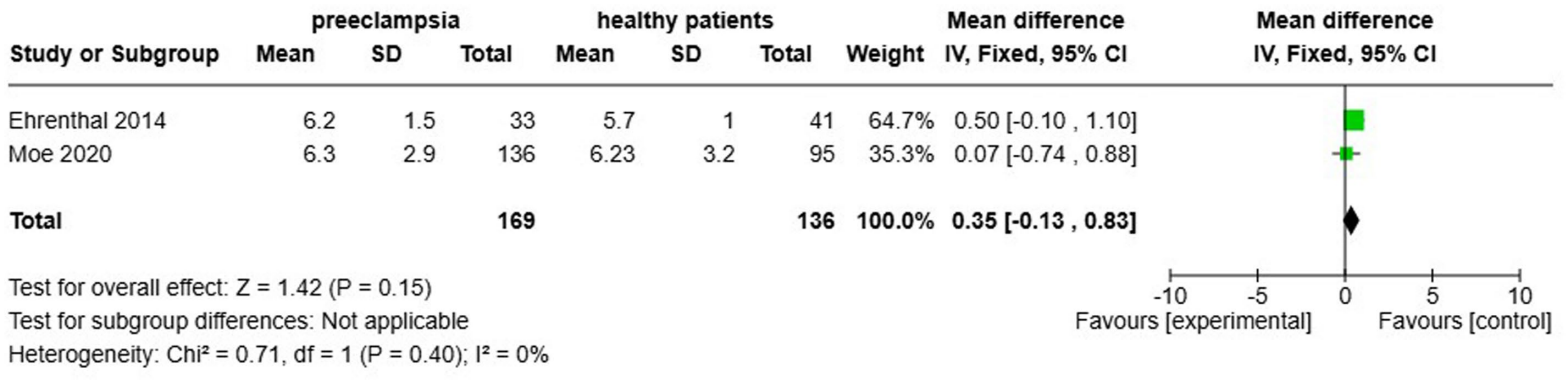
Pooled mean difference of the PWV of women with composite hypertensive disorders of pregnancy and normotensive pregnant women.

Two studies, Ehrenthal et al. (22) and Moe et al. (23) analysed cfPWV in the context of HDP, reporting no significant increase in cfPWV among affected women (MD 0.35, 95% CI [−0.13–0.83]), with zero statistical heterogeneity noted (Figure 9). This part of the analysis comprised 305 individuals (169 with HDP and an equivalent number of normotensive controls).

### Comparison of augmentation index and pulse wave velocity among women with early onset preeclampsia and normotensive women

**Figure 10:**
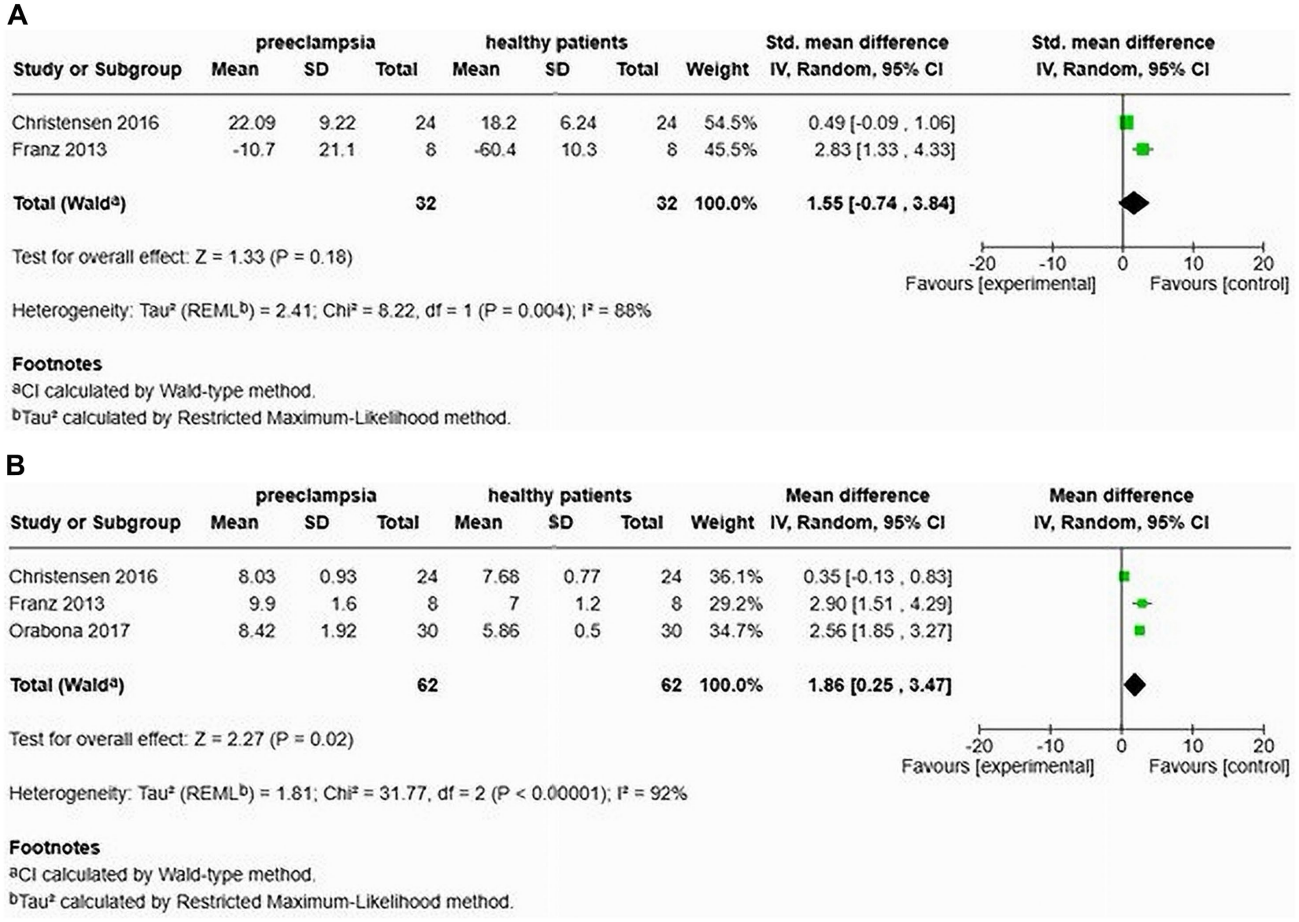
Pooled mean difference of the AIx **(A)** and PWV **(B)**, of women with early onset preeclampsia compared to normotensive women.

In the context of early onset preeclampsia (EOP), two studies, conducted by Christensen et al. (26) and Franz et al. (24), evaluated the AIx and found no significant increase associated with EOP (MD 1.55, 95% CI [−0.74–3.84]), with a heterogeneity of 88% (Figure 10A). The studies included a total of 64 participants, comprising 32 EOP and 32 normotensive women. Orabona et al. (25) was the only study to evaluate AIx after adjusting for heart rate, and it indicated a significant elevation in AIx following this adjustment (MD 0.94, 95% CI [2.17–3.70]).

Additionally, Christensen et al. (26), Franz et al. (24), and Orabona et al. (25) also assessed cfPWV in patients with EOP. The results indicated a significant increase in cfPWV (MD 1.86, 95% CI [0.25–3.47]) and a heterogeneity of 92%. A total of 124 participants were included in these studies, consisting of 62 EOP and 62 normotensive women (Figure 10B).

### Comparison of augmentation index and pulse wave velocity among women with late onset preeclampsia and normotensive pregnancy

**Figure 11:**
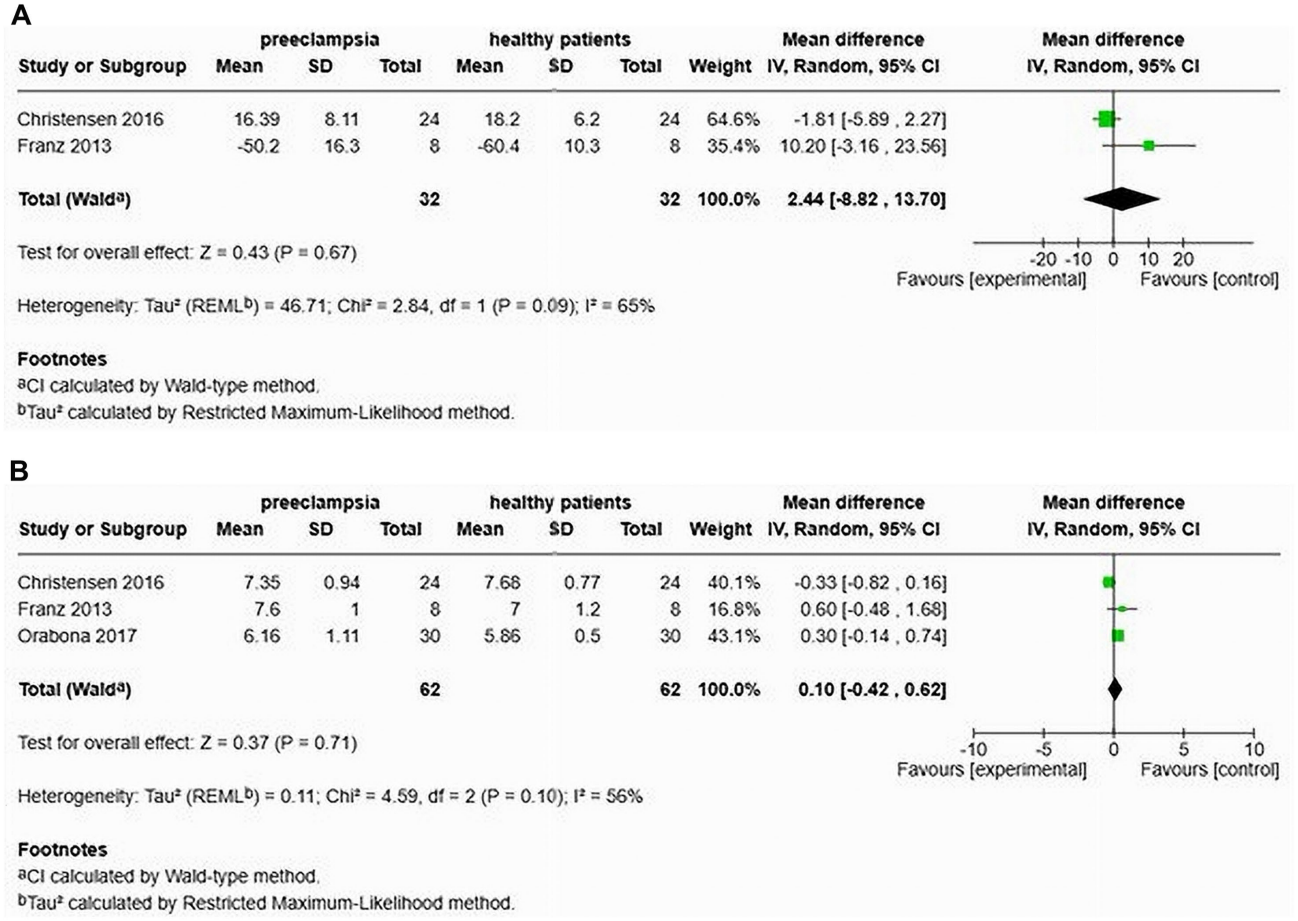
Pooled mean difference of the AIx **(A)** and PWV **(B)**, of women with late-onset preeclampsia compared to normotensive women.

Christensen et al. (26) and Franz et al. (24) also studied the AIx in cases of late-onset preeclampsia. Their analysis showed no significant difference (MD of 2.44, 95% confidence interval [−8.82–13.70]) and indicated a heterogeneity of 65% (Figure 11A). This study included a total of 64 participants, comprising 32 individuals with LOP and 32 normotensive controls. However, after adjusting for heart rate, Orabona et al. (25) found that the augmentation index was significantly higher in the LOP group (MD of 4.80, 95% confidence interval [0.60–9.00]).

Furthermore, Christensen et al. (26), Franz et al. (24), and Orabona et al. (25) also assessed cfPWV in patients with LOP. Their findings indicated no significant increase in cfPWV (MD 0.10, 95% CI [−0.42–0.62]), with a heterogeneity of 56% (Figure 11B). The total sample size in this analysis was 124 participants, including 62 with LOP and 62 normotensive individuals.

### Comparison of augmentation index and pulse wave velocity among women with HDP and normotensive pregnancy in cohort studies

**Figure 12:**
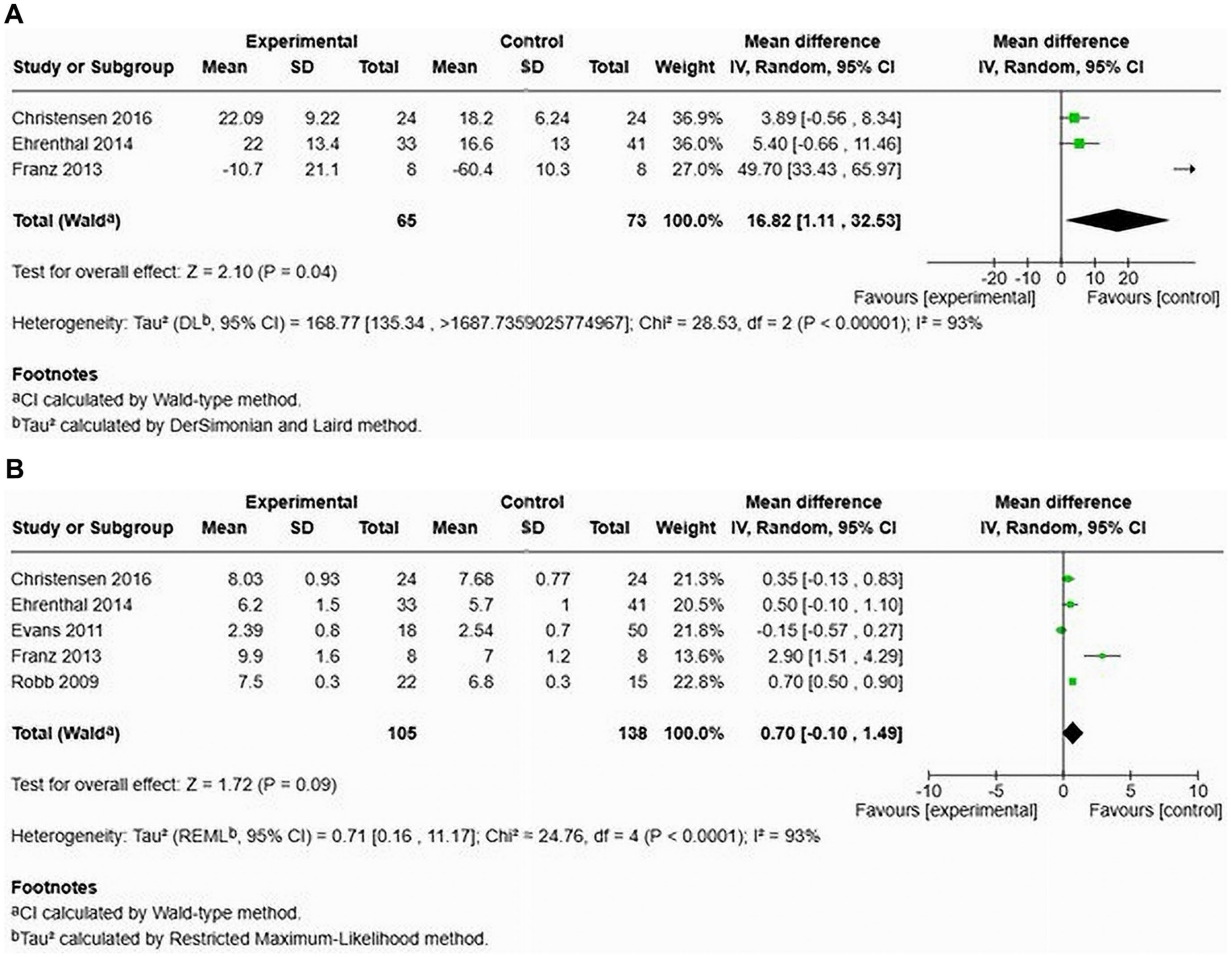
The pooled mean difference of the AIx **(A)** and PWV **(B)**, of women with HDP and normotensive women in cohort studies.

A total of three studies assessed the AIx in patients with HDP compared to normotensive women (22, 24, 26) (see Figure 12A). The pooled data demonstrated a significantly elevated AIx among women with HDP, indicating a MD of 16.82 and a 95% CI of [1.11–12.92]. This analysis included 138 participants, comprising 65 women with HDP and 73 normotensive controls. The heterogeneity among the studies was high at 93%, suggesting varying results across the studies. In the only study that adjusted the AIx for heart rate (AIx@75), the augmentation index remained significantly elevated after adjustment, with a MD of 6.40 and a 95% CI of [0.52–12.28] (22).

Furthermore, an examination of cfPWV across five studies showed no significant increase in cfPWV among women with HDP, demonstrating a MD of 0.70 with a 95% CI of [−0.10–1.49] (see Figure 12B). The heterogeneity was again high at 93%, and this analysis involved 243 patients (105 with HDP and 138 normotensive).

### Comparison of augmentation index and pulse wave velocity in women with HDP versus normotensive women in cross-sectional studies

**Figure 13:**
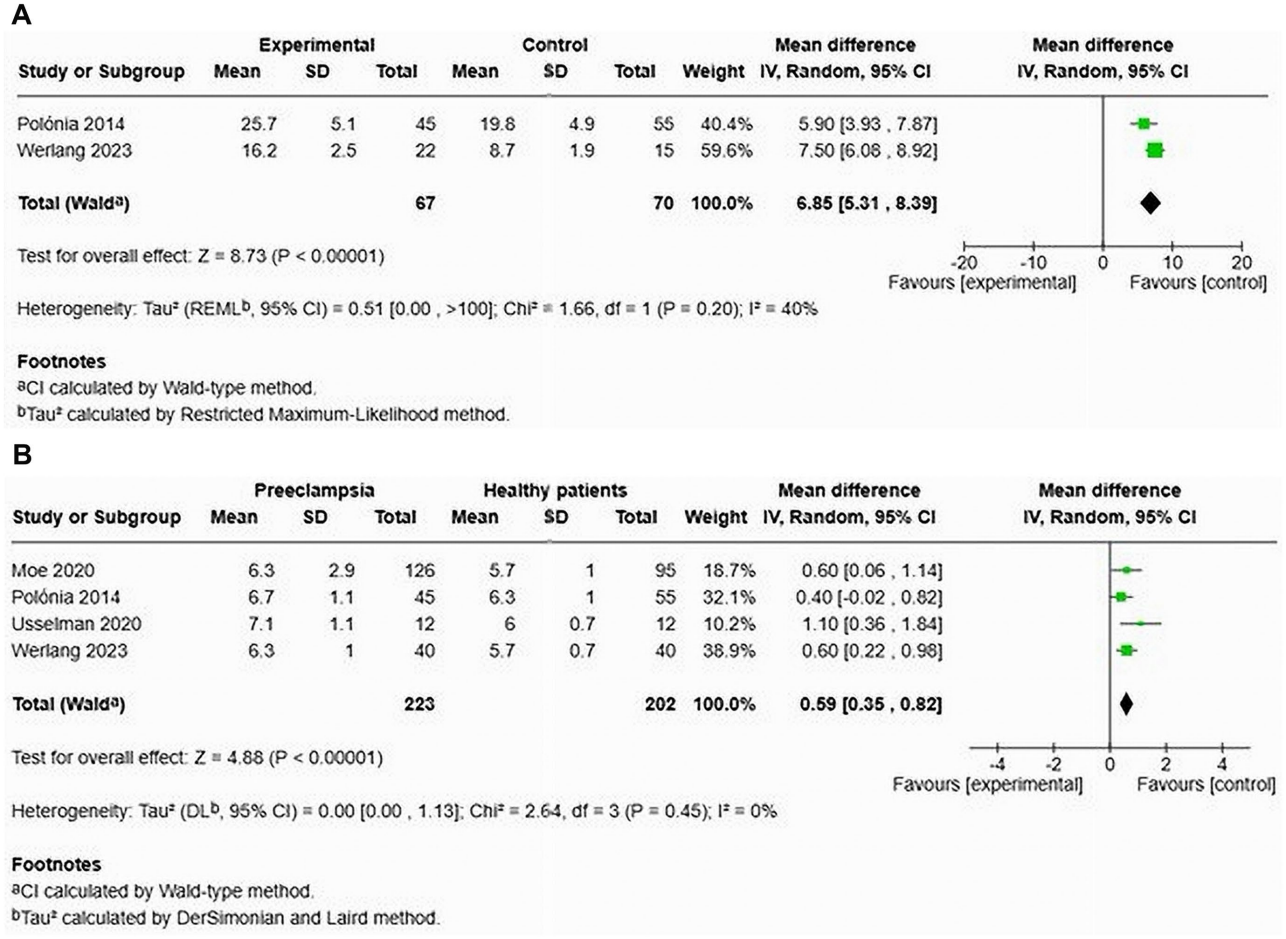
Pooled mean difference of the AIx **(A)** and PWV **(B)** of women with HDP and normotensive pregnant women in cross-sectional studies.

Two studies focused on AIx in patients with HDP compared to normotensive controls (12, 27) (see Figure 13A). The results indicated a significant increase in AIx for women with HDP, with a MD of 6.85 and a 95% CI of [5.31–8.39]. The heterogeneity among these studies was low at 40%. This analysis included a total of 137 participants, with 67 diagnosed with HDP and 70 who were normotensive. In one study, the AIx remained significantly elevated after adjusting for heart rate, with a MD of 7.50 and a 95% CI of [0.68–8.92] (12).

Four studies analysed cfPWV in the context of HDP, reporting a significant increase in cfPWV among affected women, with a MD of 0.59 and a 95% CI of [0.35–0.82] (12, 23, 27, 28). No statistical heterogeneity was observed in this analysis (see Figure 13B). This part of the analysis comprised 305 individuals (223 with HDP and 203 normotensive controls).

## Discussion

The systematic review presents a comprehensive analysis of the literature regarding arterial stiffness in women with previous history of HDP, particularly pre-eclampsia. Most studies revealed elevated arterial stiffness after 6 weeks post-delivery in women with a history of preeclampsia, suggesting ongoing vascular dysfunction that could lead to an increased cardiovascular risk lingering even years after pregnancy (29). The duration of the studies ranged from 7 weeks postpartum to 10 years post-pregnancy, showcasing the long-term implications of preeclampsia on cardiovascular health. However, not all studies demonstrated a consistent association between HDP and increased arterial stiffness. For example, a Danish study by Christensen et al. and research by Evans et al. in the United States reported no increase in arterial stiffness as measured by cfPWV and AIx (26, 30). This indicates that, while a relationship may exist, its impact can vary based on the demographic and clinical characteristics of the populations studied.

The AIx, particularly after adjusting for heart rate, was found to be elevated in women with HDP (12, 25, 27, 31, 32). This includes specific types such as composite preeclampsia and both early and late onset preeclampsia, when compared to normotensive women (25). This suggests a considerable increase in arterial stiffness, which may indicate a heightened cardiovascular risk in this preeclampsia population. The high heterogeneity (87%) observed among the studies points to variability in results, potentially due to differences in study populations, methodologies, or measurement techniques. Additionally, a single study examining AIx@75 in women with composite HDP also reported a significant increase, although heterogeneity assessment was not applicable in this case (22). These findings reinforce the idea that women with various types of hypertensive disorders experience increased arterial stiffness, emphasizing the associated cardiovascular risk. The consistently elevated AIx in both cohort and cross-sectional studies further supports the notion that there is a significant alteration in arterial function among women with a history of HDP. Additionally, a review of AIx findings 1 year after delivery indicates changes in vascular function in women with a history of HDP, suggesting a lasting cardiovascular risk. Although arterial stiffness, as indicated by AIx, may not differ significantly within the first year postpartum, long-term effects of HDP could increase arterial stiffness and potentially, cardiovascular risk. Importantly, both women in their first year after delivery and those beyond that period demonstrated a significant increase in cfPWV.

Furthermore, pooled data from eleven studies comparing various hypertensive disorders in normotensive women, as well as data from eight studies comparing preeclampsia and normotensive women, revealed a significant increase in cfPWV (12, 23, 27, 28, 30–33). However, studies on cfPWV for women with composite HDP and late onset preeclampsia showed no significant increases (15, 29). This suggests that while Aix is elevated, reflecting increased arterial stiffness, cfPWV might not indicate a similar degree of change, potentially highlighting that the two measurements assess different aspects of arterial function. cfPWV directly measures the speed at which pressure waves travel through the arterial tree. A faster cfPWV indicates stiffer arteries and is more representative of large artery function. It is less influenced by peripheral changes and wave reflections from smaller arteries (34). On the other hand, AIx measures the augmentation of pressure waves in the arteries caused by reflected waves from peripheral sites (35, 36). This metric reflects not only the stiffness of the arteries but also the timing and intensity of these reflections.

Notably, the assessment of early-onset preeclampsia also revealed a significant rise in cfPWV, contrasting with late-onset preeclampsia, which showed no significant changes. This discrepancy may indicate that the early onset of preeclampsia is associated with distinct hemodynamic changes that are absent in the later onset. Furthermore, the absence of significant mean differences in cohort studies, alongside notable findings in cross-sectional studies, suggests that cfPWV may be a more variable measure influenced by various factors present in different study designs. The contrasting results warrant further investigation to understand better the relationship between cfPWV and HDP.

### Limitations

This review had a limited number of studies, which could introduce bias. Additionally, a high level of heterogeneity was observed in most group analyses. However, assessments using the NOS revealed a low risk of bias in all studies, with the GRADE framework rating the evidence as moderate to high certainty. Furthermore, the review adhered to the PRISMA-P guidelines, and a comprehensive search was performed across several databases.

## Conclusion

This systematic review and meta-analysis suggest that arterial stiffness may remain elevated for more than 6 weeks postpartum in women with a history of HDP. However, the findings should be interpreted with caution due to heterogeneity across studies and limited number of available studies. Larger and standardized longitudinal studies are needed to confirm these results. In the meantime, regular cardiovascular monitoring for these women is recommended while awaiting more conclusive evidence.

## Data Availability

The original contributions presented in the study are included in the article/supplementary material, further inquiries can be directed to the corresponding author.
